# Changes in Airway Histone Deacetylase2 in Smokers and COPD with Inhaled Corticosteroids: A Randomized Controlled Trial

**DOI:** 10.1371/journal.pone.0064833

**Published:** 2013-05-22

**Authors:** Sukhwinder Singh Sohal, David Reid, Amir Soltani, Steven Weston, Hans Konrad Muller, Richard Wood-Baker, Eugene Haydn Walters

**Affiliations:** 1 Breathe Well Centre for Research Excellence in Chronic Respiratory Disease, University of Tasmania School of Medicine, Hobart, Australia; 2 Iron Metabolism Laboratory, Queensland Institute of Medical Research, Brisbane, Australia; University Hospital Freiburg, Germany

## Abstract

The expression of HDAC2 is reported as reduced in chronic obstructive pulmonary disease (COPD). We assessed HDAC2 expression within the airways of smokers and subjects with COPD and effects of inhaled corticosteroids (ICS), using immuno-histology to contrast with previous molecular methodology.

Endobronchial biopsies (ebb) from current smokers with COPD (COPD-CS; n = 15), ex-smokers with COPD (COPD-ES; n = 17), smokers with normal lung function (NS; n = 16) and normal controls (NC; n = 9) were immunostained for HDAC2. A double-blinded, randomized, placebo-controlled 6 months intervention study assessed effects of ICS on HDAC2 in 34 COPD subjects.

There was no difference in *epithelial* HDAC2 staining in all groups. There was a significant reduction in total cell numbers in the lamina propria (LP) in COPD-CS and NS (p<0.05). LP cellularity correlated inversely with smoking history in COPD-CS (R = −0.8, p<0.003). HDAC2 expression increased markedly in NS (p<0.001); in contrast COPD-CS was associated with suppressed signal (p<0.03), while normal in COPD-ES. ICS did not affect HDAC2 cell staining.

Our findings suggest that airway HDAC2 expression is increased in the LP by smoking itself, but is reduced in COPD. Ex-smokers have normalised HDAC2 cell expression, but ICS had no effect. The paper emphasise the pit-falls of relying on molecular data alone to define airway changes.

Clinical Trial Registration Information:

**Name of registry:**

The Australian New Zealand Clinical Trials Registry (ANZCTR)

**Registry number:**

ACTRN12612001111864

## Introduction

COPD is a disease state characterized by airflow limitation that is not fully reversible, usually progressive and associated with an abnormal inflammatory response of the lung airways in response to noxious particles and gases [1]. The increased expression of inflammatory genes is regulated by acetylation of core histones around which deoxyribonucleic acid (DNA) is wound, allowing access of pro-inflammatroy transcription factors to transcription-regulatory sites. On the other hand these activated genes are switched off by at least partly by deacetylation of these histones [2]. However, the control of airway inflammation and response to airway oxidative stress is highly complex and many overlapping mechanisms are involved.

Airway inflammation in the airways responds [3] to therapeutic corticosteroids but only relatively poorly in COPD even though steroids have been shown to have some important specific beneficial effect clinically both in the short and long term [4], [5]. There is evidence that relative corticosteroids anti-inflammatory resistance in COPD may be partly due to decrease in histone deacetylase activity, and especially the type-2 enzyme (HDAC2) [6], [7]. Though exact mechanisms are still not clearly understood, it is suggested that this may involve oxidative modulation of HDACs by nitrosylation on distinct tyrosine residues in response to tobacco smoke [2].

Research regarding the role of histone acetylation and deacetylation in chronic inflammatory disease is only in its infancy, and even more so in COPD, where the picture has probably been made rather over-simplified, as indeed the degree of ICS insensitivity has been exaggerated. Inhaled corticosteroids (ICS) are used very widely in COPD clinically. However, there is a significant body of literature suggesting that expression and activity of anti-inflammatory HDAC2 are reduced in COPD lungs, airways and alveolar macrophages and becomes worse with severity of the disease [6], [7].

The methodology that has been used in previously published investigations has largely depended on molecular ribonucleic acid (RNA) quantitation and protein analyses, which does not take into account potential differences in absolute or relative cellular profiles in airway tissues in disease *versus* control groups. In these situations, biases could arise, and there has been a serious lack of comprehensive biopsy studies to confirm the extent of suppressed HDAC2 expression through the use of immunostaining techniques that provide additional information on cell numbers and type within the airways of COPD [8]. Further rationale for using immunostaining techniques is provided by studies that clearly demonstrate that ICS affect the profile of cell populations in the airways [3], [9], [10], [11], [12], [13], [14], [15]. In order to further address the potential confounder of changes in cellularity of the airway wall when interpreting HDAC2 expression in smokers and COPD, we performed both cross-sectional and longitudinal studies to test our hypothesis that the current literature is correct in terms of HDAC2 being down-regulated in the COPD airway, but that HDAC2 levels could be normalised by aggressive ICS therapy [8].

## Materials and Methods

The protocol for this trial and supporting CONSORT checklist are available as supporting information; see Checklist S1 and Protocol S1.

### Subjects and study design

Subjects were recruited by advertisement in local newspapers and placement of posters in clinic waiting areas in the hospital, as well as on the notice boards of social and Veterans clubs. Study was approved by “The Human Research Ethics Committee (Tasmania) Network” and “The Alfred Health Human Ethics Committee” Melbourne (The Alfred Hospital). All subjects gave written, informed consent prior to participation. Subjects with inability to give written informed consent were also excluded. Potential participants were interviewed and examined by a respiratory physician and subjects with a history suggestive of asthma, that is, symptoms in childhood, related atopic disorders, eczema or hay fever, significant day-to-day variability or prominent nocturnal symptoms, or a history of wheeze rather than progressive breathlessness and any who had previously used ICS were excluded. Other exclusion criteria included significant uncontrolled comorbidities such as diabetes, angina or cardiac failure, and other coexisting respiratory disorders including pulmonary fibrosis, lung cancer and bronchiectasis [3].

The diagnosis of COPD was made according to GOLD guidelines [16]. Subjects had more than 15 pack-year smoking history and subsequently obtained bronchoalveolar lavage (BAL) fluid had to be free of bacterial colonisation. COPD ex-smokers with six months of smoking cessation were included. Normal healthy volunteers had no history of respiratory illness or smoking. For normal lung function current smokers the inclusion criteria were; a minimum 10 pack-year history of cigarette smoking with spirometry within normal limits (FEV1 (forced expiratory volume)>80% of predicted, and FEV1/FVC (forced vital capacity)>70%) and no scalloping out of the expiratory descending limb of the flow-volume curve, suggesting small airway dysfunction.

**Figure 1:**
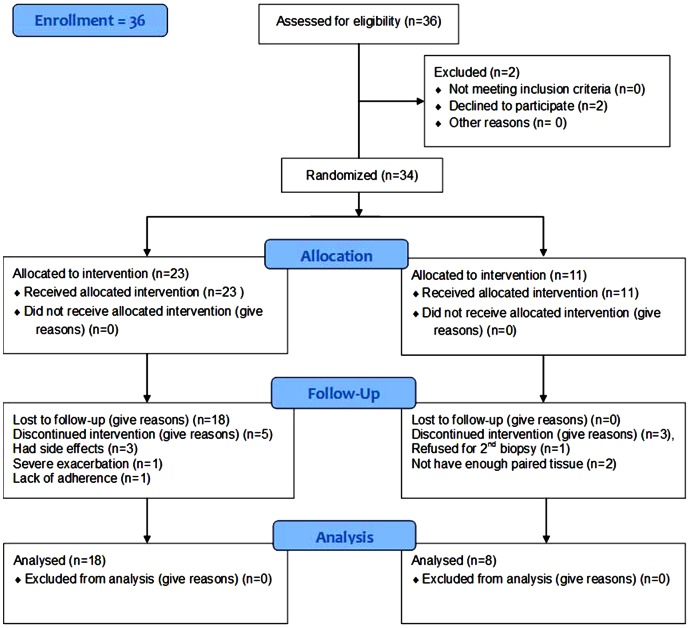
Study design. Thirty four COPD patients; two week run-in period; then bronchoscopy and airway biopsy; then patients randomized 2∶1 by research nurses into receiving fluticasone propionate or placebo for 6 months by using a computer generated random-numbers table; bronchoscopy and airway biopsy then repeated.

In the cross-sectional study, 17 current smokers with established COPD (CS), 16 current smokers with normal lung function (NS), 17 ex-smokers with COPD (ES) and 15 normal healthy, never-smoking controls (NC) were recruited by advertisement for bronchoscopy and airway biopsy (Table 1). Then, using a computer generated random numbers table, 34 COPD participants only were randomized 2∶1 to fluticasone propionate (FP) (Accuhaler; Glaxo-Wellcome, Middlesex, UK) 0.5 mg/twice daily or placebo via identical multi-dose dry powder inhaler devices in a double blinded randomised controlled trial for six months (Table 2 & Figure 1). After 6 months of treatment lung function and bronchial biopsies were performed again. This trial is registered with Australian New Zealand Clinical Trials Registry (ANZCTR: ACTRN12612001111864). All subjects gave written, informed consent prior to participation.

**Table 1:** Demographic and lung function data for subjects.

Groups(numbers)	COPD-CS(n = 17)	COPD-ES(n = 15)	NS(n = 16)	NC(n = 15)
GOLD I/GOLD II‡	10/7	8/7	N/A	N/A
Male/female	9/8	9/6	12/4	7/8
Age (years)	61 (46–78) (p = 0.001)*	62 (53–69) (p = 0.001)*	50 (30–66) (p = 0.313)	44 (20–68)
Smoking (pack years)	45 (18–78)	51 (18–150)	32 (10–57)	0
FEV1% predicted(Post BD)†	83 (66–102) (p<0.001)*	83 (54–104) (p<0.001)*	99 (78–125) (p = 0.01)*	113 (86–140)
FEV1/FVC%(Post BD)†	59 (46–68)(p<0.001)*	57 (38–68)p<0.001)*	77 (70–96)(p = 0.218)	82 (71–88)

Data expressed as median and range

* Significance difference from NC

† Post BD values after 400 µg of salbutamol****

‡ Diagnosis of COPD was made according to GOLD guidelines [16]

**Table 2:** Demographic and lung function data for COPD subjects in the intervention study.

Groups (numbers)	FP (22)	Placebo (10)
GOLD I/GOLD II†	10/12	4/6
Male/female	8/14	3/7
Age (years)	60(46–69)	62(52–69)
COPD-CS/COPD-ES	12/10	3/7
Smoking (pack years)	42(18–150)	54(22–147)
FEV1% predicted (Post BD)*	77(55–112)	77(54–94)
FEV1/FVC% (Post BD)*	58(41–66)	56(38–67)

There were no significant differences between groups in demographics, lung function or anatomical indices of interest before intervention (cellularity or remodelling).

Data expressed as median and range

* Post BD values after 400 µg of salbutamol****

† Diagnosis of COPD was made according to GOLD guidelines [16]

### Bronchoscopy

Bronchoscopy was performed using standard techniques. Briefly, subjects were pre-medicated with nebulized salbutamol (5 mg) 15–30 min before the procedure. Sedation was achieved with intravenous midazolam (3–10 mg) and fentanyl (25–100 µg). Lignocaine (4%) was used for topical anaesthesia above the vocal cords and 2% lignocaine was used to anaesthetize the airways below the cords, in 2 ml aliquots as required, up to a maximum of 6 ml. Subjects were monitored by pulse oximetry throughout the procedure and oxygen was administered to all subjects at a flow rate of 4 L/min [17]. Eight biopsies from secondary carina of segmental and sub-segmental bronchi in the right lower lobe were obtained. There were no complications from the procedures.

### Immunostaining

Bronchial biopsy sections were immunostained for HDAC2 using monoclonal antibody: anti-HDAC2 (Abcam cat no. ab12169, clone hdac2-62 at 1∶4000 for 1 hour at room temperature), together with a horseradish peroxidase (HRP) conjugated DAKO Envision plus reagent for secondary antibody binding and colour resolution using diaminobenzidine (DAB). In each case a non-immune IgG1 negative control (Dakocytomation, Denmark X0931 clone DAK-GO1) was performed to eliminate false positive staining.

### Biopsy analysis

Computer-assisted image analysis was performed with a Leica DM 2500 microscope (Leica Microsystems, Germany), Spot insight 12 digital camera and Image Pro V5.1 (Media Cybernetics, USA) software. Using the image analyser HDAC2 positive and total numbers of cells were counted up to 50μ deep into the lamina propria and results presented as cells per mm^2^ of lamina propria. In the epithelium HDAC2 was measured as percentage of epithelium stained for HDAC2 over total basement membrane length. All slides were coded and randomised by an independent person (SW) and then counted in a single batch by a single experienced observer (SS) with quality assurance on randomly selected slides provided by a professional academic pathologist (HKM).

### Statistical analysis

The distributions were generally skewed so results are presented as medians and ranges and non-parametric analyses of variance were performed (a non-parametric ANOVA, Kruskal Wallis Test comparing medians across all the groups of interest), and specific group differences then explored using the Mann Whitney U test using adjusted p value. The results are presented as scatter plots. Wilcoxon two related-samples test was used to test the effect of ICS and placebo in the longitudinal study. At the time of developing this study there were no data to base power calculations on, and so we relied on previous precedent in other studies and what was practicable in terms of recruitment, this study from our group suggested that approximately 15 subjects would be an optimal number with little advantage in increasing beyond this. [18]. Associations between variables were assessed using Spearman's rank test. Statistical analyses were performed using SPSS 15.0 for Windows, 2003, with a two-tailed *P*-value≤0.05 being considered statistically significant.

## Results

The group demographics of subjects who participated in the study are presented in Table 1 and 2. Details of randomization are given in Figure 1.

### HDAC2 expression in the airway epithelium

**Figure 2:**
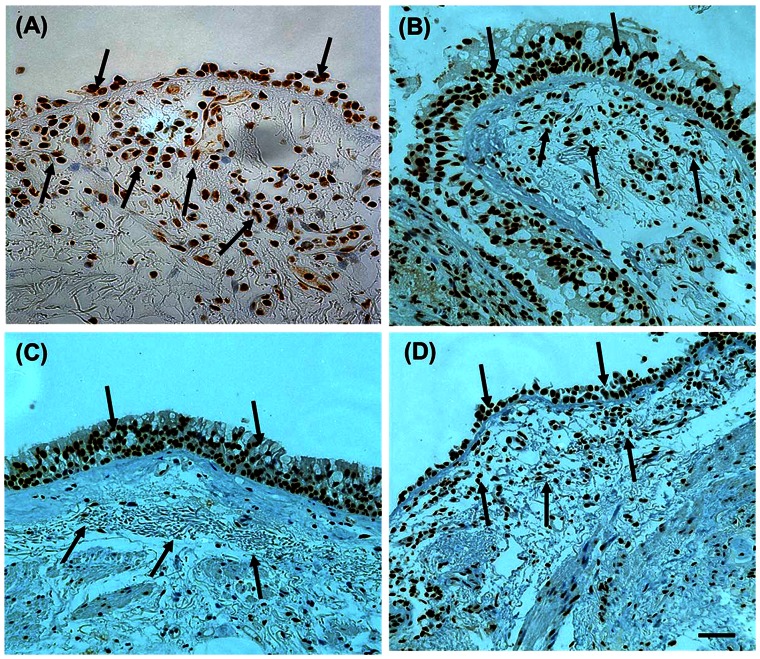
Bronchial biopsy sections stained for HDAC2. (**A**) normal control; (**B**) normal lung function smoker; (**C**) COPD current smoker; and (**D**) COPD ex-smokers: black arrows indicate the brown staining of HDAC2 positive cells in the epithelium and in the LP, and also *indicating decreased cellularity in the LP in (B) and (C)*. Original magnification, x400. Scale bar = 50 µm.

**Figure 3:**
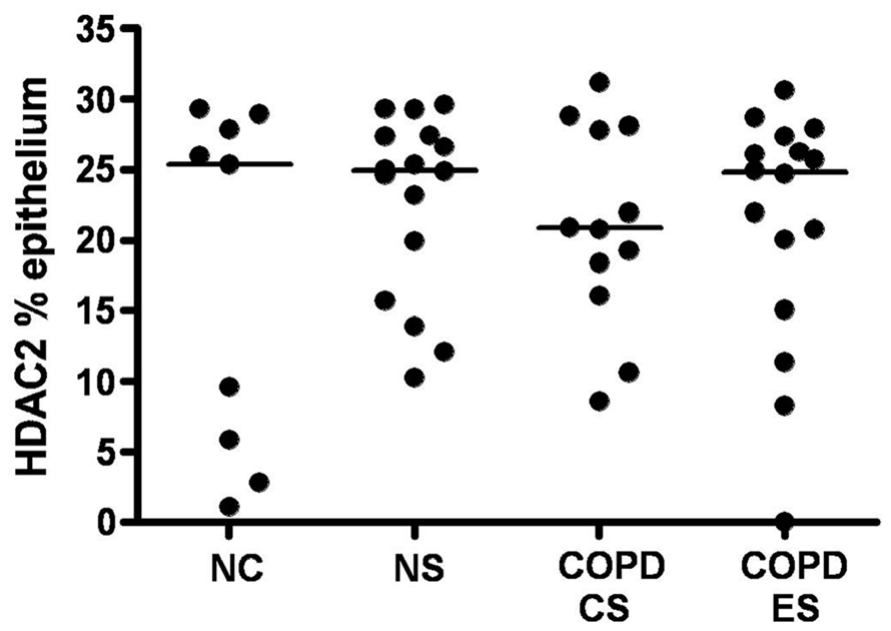
HDAC2 staining in the epithelium. Percentage area of epithelium stained for HDAC2 in COPD current smokers (COPD-CS) and ex-smokers COPD-ES), normal lung function smokers (NS) compared to normal controls (NC) with no significant difference between groups, but with a great deal of between subject variability. Horizontal bars represent the median for each group.

HDAC2 expression in the airway epithelium (as measured by percentage area of epithelium stained for HDAC2) was not significantly different between the groups (Figure 2 and 3), [median (range); NC 25.3% (1.1%–29.3%); NS 24.9% (10.2%–29.6%); COPD-CS 20.8% (8.6%–31.2%); COPD-ES 24.7% (0%–30.6%) p = 0.7], although it was reduced on average in the current smoking COPD but with wide inter-subject scatter. ICS made no difference to HDAC2 staining [median (range); active arm 23.3% (8.6%–31.2%) before *versus* 19.6% (1.6%–41.5%) after treatment, p = 0.2; placebo, 20.7% (0%–30.6%) before *versus*, 20.7% (3.7%–26.3%) after treatment, p = 0.5].

### Total cellularity in the lamina propria

**Figure 4:**
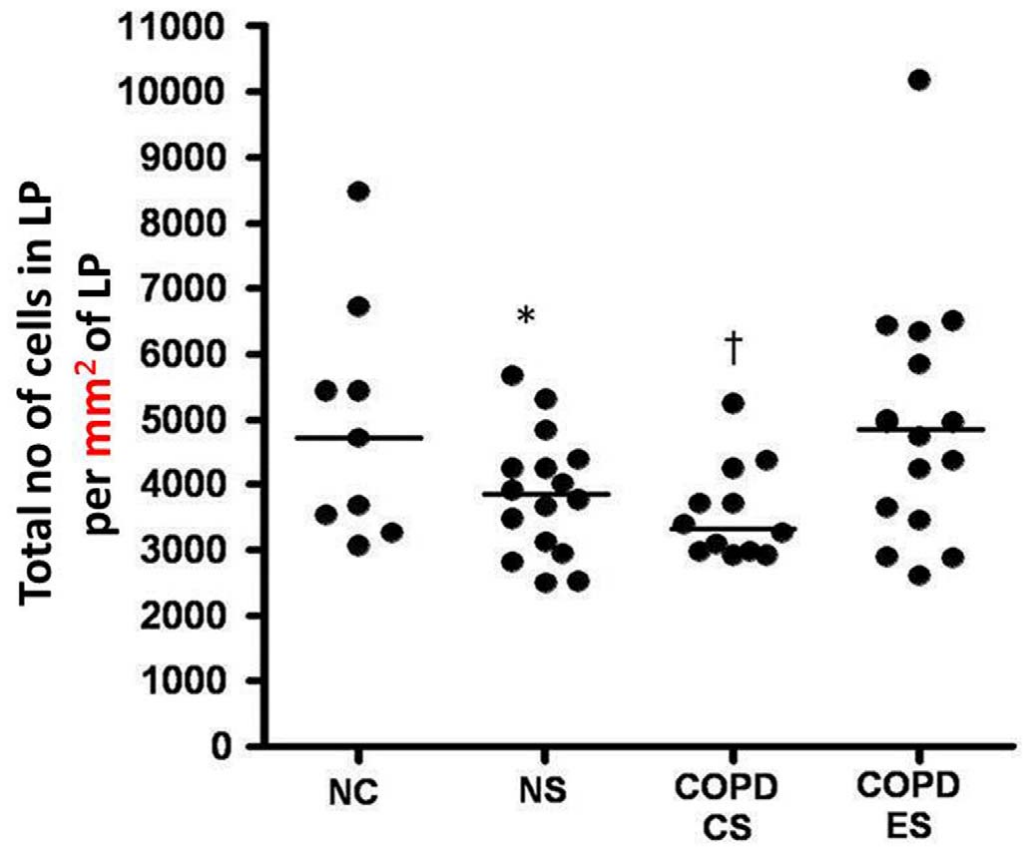
Lamina propria cellularity. Total number of cells in the lamina propria per mm^2^ of lamina propria; *significant difference from NC and COPD-ES (p<0.05); †significant difference from NC and COPD-ES (p<0.03).

**Figure 5:**
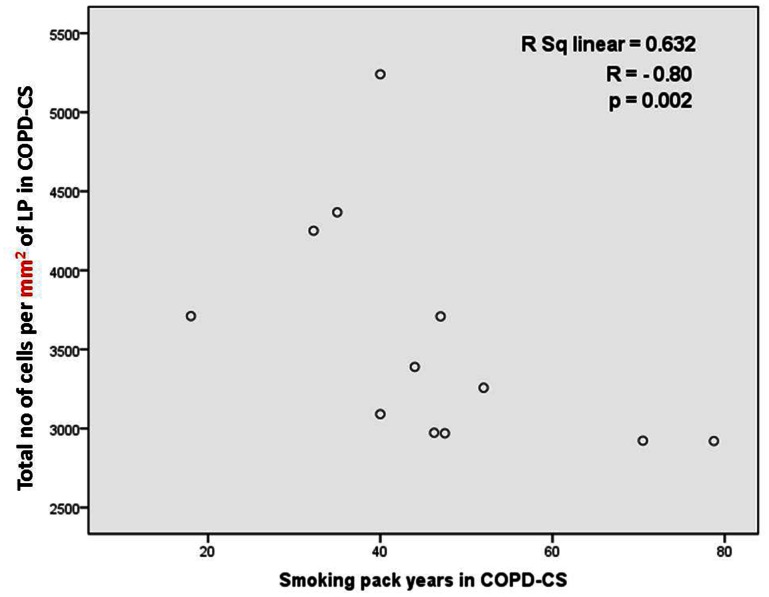
Relationship between total number of cells and smoking history. Correlation between total number of cells per mm^2^ of lamina propria in COPD-CS and smoking (pack years).

Total cell numbers were significantly reduced in both current smoker groups, [median (range); NC, 4706.9 per mm^2^ (3065.2 per mm^2^–8475.4 per mm^2^) *versus* NS, 3839.4 per mm^2^ (2495 per mm^2^–5668.1 per mm^2^); COPD-CS, 3323.2 per mm^2^ (2920.4 per mm^2^–5240.2 per mm^2^) p<0.05] (Figure 2 and 4). This total cell change was strongly and negatively correlated with smoking history in COPD-CS (R = −0.8, p<0.003) (Figure 5). COPD-ES were significantly different from COPD-CS [median (range); COPD-ES, 4958.9 per mm^2^ (2606.1 per mm^2^–10178.5 per mm^2^) *versus* COPD-CS, 3323.2 per mm^2^ (2920.4 per mm^2^–5240.2 per mm^2^) p<0.03), with total cellularity being essentially normal. ICS made no difference to total number of cells in the lamina propria [median (range); active arm, 3583.3 per mm^2^ (2606.1 per mm^2^–5834.2 per mm^2^) before *versus* 3633.5 per mm^2^ (2591.5 per mm^2^–6781.6 per mm^2^) after treatment, p = 0.5; placebo, 4364 per mm^2^ (2920.4 per mm^2^–10178.5 per mm^2^) before *versus* 4097.5 per mm^2^ (2895.5 per mm^2^–7936.3 per mm^2^) after treatment p = 0.8].

### Absolute HDAC2 staining in the lamina propria

**Figure 6:**
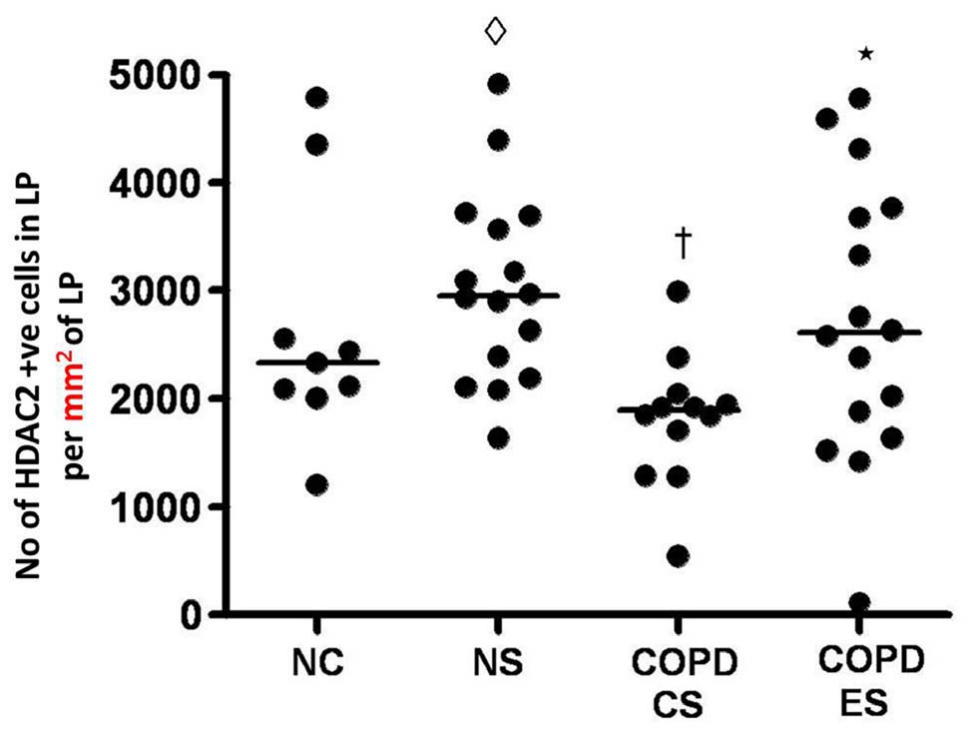
HDAC2 staining in the lamina propria. Number of HDAC2 positive cells in the lamina propria per mm^2^ of lamina propria; †significant difference from NC (p<0.03); ◊significant difference from COPD-CS (p<0.001); *significant difference from COPD-CS (p<0.05).

Compared to NC there was a significant reduction in HDAC2 positive cells in the lamina propria in COPD-CS [median (range); NC, 2341.2 per mm^2^ (1213.1 per mm^2^–4797.1 per mm^2^) *versus* COPD-CS, 1894.2 per mm^2^ (550.5 per mm^2^–2997.2 per mm^2^) p<0.03]. Un-expectedly, normal lung function smokers had significantly more HDAC2 positive cells in the lamina propria [median (range); NS, 2952.4 per mm^2^ (1647.4 per mm^2^–4918.8 per mm^2^) *versus* COPD-CS, 1894.2 per mm^2^ (550.5 per mm^2^–2997.2 per mm^2^) p<0.001] and also compared to normal controls although not statistically significant. There were significantly more HDAC2 positive cells in COPD-ES compared to COPD-CS [median (range); COPD-ES, 2611.5 per mm^2^ (122 per mm^2^–4786.3 per mm^2^) *versus* COPD-CS, 1894.2 per mm^2^ (550.5 per mm^2^–2997.2 per mm^2^) p<0.05] (Figure 2 and 6); ie COPD-ES smokers were essentially normal. No association was found between quantitative HDAC2 staining and lung function measurements in any group. ICS made no difference to HDAC2 positive cell numbers, [median (range); active arm, 1944.4 per mm^2^ (550.5 per mm^2^–4599.7 per mm^2^) before *versus* 1914.5 per mm^2^ (515.2 per mm^2^–5038.9 per mm^2^) after treatment, p = 0.5; placebo, 2159.7 per mm^2^ (122 per mm^2^–4320.6 per mm^2^) before *versus* 1442.3 per mm^2^ (542.3 per mm^2^–3138.4 per mm^2^) after treatment p = 0.3].

### Percentage cell HDAC2 staining in the lamina propria

**Figure 7:**
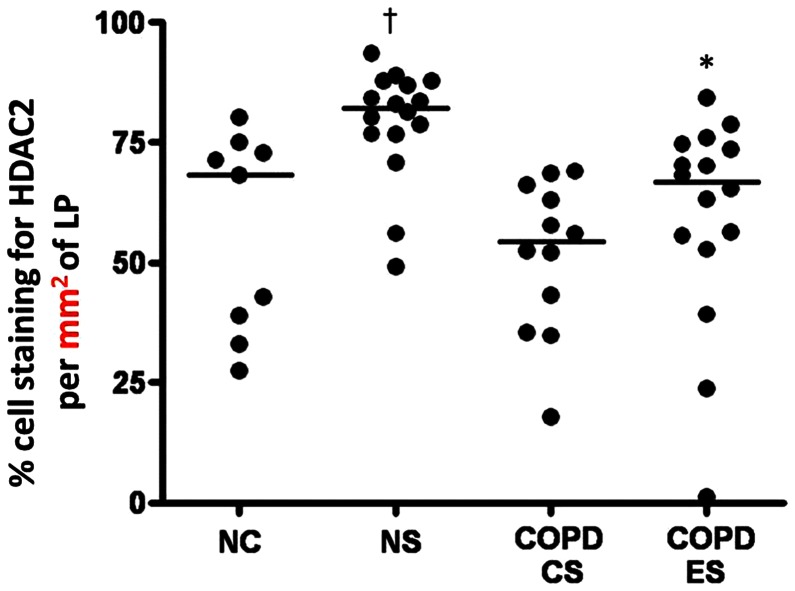
HDAC2 percentage staining in the lamina propria. Percentage cell staining for HDAC2 in the lamina propria per mm^2^of lamina propria; †significant difference from COPD-CS, ES and NC (p<0.001); *significant difference from COPD-CS (p<0.03).

For percentage of LP cells staining for HDAC2 (i.e. taking into account individual, smoking, and disease-related changes in total cell numbers), the picture closely resembled that for absolute cell data. Thus, NS were significantly different from all other groups [median (range); NS, 82.1% (49.1%–93.5%) *versus* NC, 68.2% (27.6%–80.1%), COPD-CS, 54.3% (17.8%–69%), COPD-ES, 66.8% (1.20%–84.3%) p<0.001], with smoking apparently stimulating HDAC2 expression. In COPD-CS HDAC2 expression was reduced compared to normal controls, but not significantly so. COPD-ES were significantly different from COPD-CS [median (range); COPD-ES, 66.8% (1.20%–84.3%) *versus* COPD-CS, 54.3% (17.8%–69%) p<0.03] but quite similar to NC [median (range); COPD-ES, 66.8% (1.20%–84.3%) *versus* NC, 68.2% (27.6%–80.1%) p = 0.42] (Figure 7). Again, no associations were found with lung function measurements in any group cross-sectionally, and percentage cell HDAC2 staining did not change with ICS treatment [median (range); active arm, 54.4% (17.8%–78.8%) before *versus* 51.7% (15.3%–75.6%) after treatment, p = 0.5; placebo, 64.5% (1.20%–84.3%) before *versus* 32.2% (8.6%–79.8%) after treatment p = 0.8].

## Discussion

To the best of our knowledge, this is the first detailed airway biopsy immunostaining study of HDAC2 expression in COPD and its potential reversibility with ICS or/and smoking cessation (albeit the latter in a cross-sectional group-comparison only at this stage). The airway epithelium showed strong HDAC2 expression, but this was not significantly different between the groups, although COPD current smokers showed a slight decrease in average HDAC2 staining, which mirrored that found in the LP. The difference between groups, even if real (and probably confounded by a likely Type-II statistical error due to limited numbers), hardly suggests that this is a major effect with HDAC2, with more variability observed between individuals than between groups. Indeed, the main message may be that HDAC2 expression in the epithelium was pretty well preserved generally in smokers, suggesting that there is sufficient HDAC2 expression here to allow ICS to be effective in this compartment (as indeed we have shown in subsequent work, data not shown). Further, we found no change in epithelial HDAC2 staining with ICS therapy.

In the LP the data were complex but striking. We found that there was a decrease in the total number of cells in the LP in current smokers, both with normal lung function and with COPD, compared to normal controls. There has been no previous differentiation between the hyper-cellularity around the reticular basement membrane [19], [20] and the hypo-cellular underlying layer. Interestingly, though, there are published micrographs from previous published studies of smokers and COPD subjects that illustrate identical changes to the one we are describing, but without formal quantitation [6]. Notably, normal lung function smokers also showed a decrease in total cellularity whilst the COPD-ES group had relatively normal total cellularity suggesting that smoking cessation had “normalised” this component of effects of smoking. What seems clear is that changes in airway cellularity, both in absolute and differential terms, need to be taken into account when interpreting quantitative PCR data for biopsy material, or confounding of the data and/or its interpretation is likely.

Our data for HDAC2 positive cells in the LP suggest that HDAC2 positive cells increase in both absolute and percentage terms in smoking per-se, but in contrast are decreased in current smokers with COPD. On the other hand there was an increase in HDAC2 positive cells in the lamina propria of COPD ex-smokers compared to COPD current smokers; essentially returning to normal. The data on percentage cell staining for HDAC2 in the LP are especially pertinent, as this takes into account potential confounding by changes in absolute number of cells. The general picture, however, remained much the same as for the absolute cell data. Overall, and especially given the relatively small number of individuals per group, the results quite strongly suggest that smoking itself generally stimulates anti-inflammatory HDAC2 expression, but is either modified by the COPD process or is exposing a group of individuals which responds differently to cigarette smoke without an anti-inflammatory HDAC2 response and is therefore vulnerable as a result to developing airway remodelling and fixed obstruction. Thus, there does seem a dichotomy between smokers who do or do not have COPD as suggested by Barnes et al, and our data is consistent with their suggestion that switching HDAC2 on or off may be the key to different outcome. Furthermore, ICS made no difference to percentage cell HDAC2 staining in COPD, so this is not a mechanism by which steroids offer some protection in the natural history of COPD.

Compared to the effects of ICS, quitting smoking may well have a potential for up-regulating HDAC2 at a cell level as shown by percentage increase in HDAC2 staining in the LP of COPD ex-smokers. Much of the effect seems related to change in total cell numbers but percentage cell staining also recovered compared to the actively smoking COPD group. We now need long term smoking cessation studies to confirm and tease out these findings.

The key finding of an increase in anti-inflammatory HDAC2 expression in the LP in normal lung function smokers but a decrease in those who have developed COPD, is also generally true for HDAC2 expression in the epithelium as well., although changes are smaller and would seem to be less biologically significant.

We cannot say at this stage which particular cell type(s) is decreased in the LP in smokers, or why, nor which cells are expressing HDAC2. For more information we need double staining studies for different cells in the lamina propria ie HDAC2 plus specific cell type markers. It has been suggested that in COPD there is increased cell apoptosis [21], [22], which could perhaps be related to the finding of hypo-cellularity. Although, this is a controversial area, and is not clearly established, increased oxidative stress produced by tobacco smoke and protease–antiprotease imbalance, and also genetic susceptibility, may contribute to increased apoptosis in COPD airways [23], [24]. In a recent study published by our group [25] we found that there is a significant reduction in total number of vessels in the lamina propria in smoking/COPD, so decreased vascular supply might be a contributing factor to decreased cellularity in COPD airways or there may be another common underlying cause of hypo-cellularity and hypo-vascularity below the reticular basement membrane. This deserves further study.

In summary, our data suggest that HDAC2 expression is increased in physiologically normal smokers but reduced in current smokers with COPD, though the latter finding is partly confounded by general decrease in cellularity in the LP. Quitting smoking may well have a real effect on up-regulating HDAC2 at a cell level, but it is not affected by ICS therapy. We need further comprehensive double-staining immunohistochemical studies to fully understand cellular changes in the LP and prospective long term smoking cessation studies to confirm these specific findings. Molecular methods, as exclusively published in the past on this topic of HDAC2 in the airways, cannot fully take account of such changes in the cellular environment[26] and cannot be interpreted on simple face value. However, the proposition that HDAC2 activity is decreased in the airways in COPD does seem to be correct, with major implications for understanding the aetiology of this common disease.

## Supporting Information

Checklist S1 **CONSORT checklist.**
(DOC)

Protocol S1 **Trial protocol.**
(PDF)
