# Supplementary material for: Changes in Airway Histone Deacetylase2 in Smokers and COPD with Inhaled Corticosteroids: A Randomized Controlled Trial
Source: PLoS One. 2013 May 22;8(5):e64833. doi: 10.1371/journal.pone.0064833 (PMC3661479; doi:10.1371/journal.pone.0064833)
Supplement: Protocol S1 — Trial protocol. (PDF) [file pone.0064833.s002.pdf]

Research and Development Office
GPO Box 252-01
Hobart, Tasmania 7001 Australia
Tel: 03 62262763 Fax: 03 62267148
Email: Human.Ethics@utas.edu.au

UNIVERSITY OF TASMANIA

HUMAN RESEARCH ETHICS COMMITTEE

APPLICATION: INVESTIGATION INVOLVING HUMAN SUBJECTS

INVESTIGATION NUMBER (Office use)

TITLE of proposed investigation [i]

Effects of the inhaled corticosteroid (ICS) fluticasone on Pulmonary and Airway inflammation in Chronic Obstructive Pulmonary Disease (COPD).

A. OUTLINE OF PROPOSAL

Applicants [ii]

Title/Name	Position	School or Discipline
Prof E Haydn Walters	Professor of Medicine	Medicine
Dr Richard Wood-Baker	Senior Leclurer in Medicine	Medicine

Contact details for chief investigator

'Phone 6226 4870 Fax 6226 4894 Email Haydn.Walters@utas.edu.au

Purpose [iii]
Research

Aims [iv]
To describe the range of airway inflammatory infiltrate in a group of patients with mild to moderate COPD, and to relate that cell picture to the response to ICS therapy over a 6 month period.

The Hypothesis is that positive benefit of ICS therapy in COPD patients is most marked in those with the most prominent degree of airway eosinophilia.

Justification [v]
This study commenced in Melbourne (Alfred Hospital) in October 2000, and by the end of 2001 we are on schedule to have recruited 20 participants. We are aiming to study approx. 36 individuals in total to give what we believe will be adequate power to detect differences between treatment groups in airway inflammatory markers. We are looking to recruit a further 16 COPD sufferers in Hobart over the first half of 2002.

Thus the specific aim of this application is to allow completion of a study that Prof Walters started in Victoria, and now wishes to transfer to Tasmania. From a scientific and clinical perspective the study will give significant insights into the patho-physiology of COPD and the effects of ICS on the patients.

**Period of investigation** [vi]			
Commencement date	1.1.2002	Completion date	31.12.2002 for the clinical part of the study.

Funding [vii]
Source/potential source of funding and amount:
£ sterling 67.5 K (approx. $180K A) from Glaxo Smith Klein: (approx. $20K will need to go to the Alfred to pay staff continuing to work on the project there.

Do the investigators have any financial interest in this project? NO

Review of ethical considerations [viii]
Has this protocol previously been submitted to the University Ethics Committee? NO

Does this project need the approval of any other Ethics Committee? YES
The Alfred Hospital Ethics Committee, Melbourne.
If 'YES', what is its current status?
The study is still ongoing at the Alfred (see enclosed certificate). Recruitment there will end at the end of 2001 with final bronchoscopy in approx. June 2002. Because of Prof Walters move to Hobart it will be logistically easier to complete the study here with all recruits beyond the end of 2001.

Relevant references
List references
(a) by the investigator; The References from Prof Walters work are to emphasise experience in such bronchoscopically based studies in airway disease. References attached.
(b) by others:-

1. Thompson AB, Mueller MB, Heires AJ et al. Aerosolised beclomethasone in chronic bronchitis. Improved pulmonary function and diminished airway inflammation. Am Rev Respir Dis 1992; 146: 389-95.
2. Saetta M, Turanto G, Facchini FM et al. Inflammatory cells in the bronchial glands of smokers with chronic bronchitis. Am J Crit Care Med 1997; 156: 1633-9.
3. O'Shaughnessy TC, Ansari TW, Barnes NC, Jeffery PK. Inflammation in bronchial biopsies of subjects with chronic bronchitis: inverse relationship of CD8+ lumphocytes with FEV1. Am J Respir Crit Care Med 1997; 155:852-7.
4. DiStephano A, Capelli A, Lusuardi M et al. Severity of airflow limitation is associated with severity of airway inflammation in smokers. Am J Resp Crit Care Med 1998; 158:1277-85.
5. Sun G, Stacey MA, Vittori E et al. Cellular and molecular characteristics of inflammation in chronic bronchitis. Eur J Clin Invest 1998; 28:314-72.
6. Jeffery PK. The value of bronchial biopsies: assessment of inflammation in asthma and COPD. Eur Resp Rev 1998; 8:1079-1085.
7. Saetta M, DiStefano A, Maestrelli P, et al. Airway eosinophilia in chronic bronchitis during exacerbations. Am J Respir Crit Care Med 1994; 150:1646-52.
8. Balzamo G, Stefanelli F, Iorio C et al. Eosinophil inflammation in stable chronic bronchitis during exacerbations. Am J Respir Crit Care Med 1999; 160: 1486-92.
9. Burge PS. EUROSCOP, ISOLDE and the Copenhagen City Lung Study. Thorax 1999; 54:287-8.
10. Pauwels RA, Lofdahl C-G, Laitinen LA et al. Long-term treatment with inhaled budesonide in persons with mild chronic obstructive pulmonary disease who continue smoking. N Engl J Med 1999; 340:1948-53.
11. Paggiaro PL, Dahle R, Bakran I et al. Multicentre randomised placebo-controlled trial of inhaled Fluticasone propionate in patients with chronic obstruction pulmonary disease. Lancet 1998; 351:773-80.
12. van Grunsven PM, van Schaych CP, Derenne JP et al. Long term effects of inhaled corticosteroids in chronic obstructive pulmonary disease: a meta-analysis. Thorax 1999; 54: 7-14.
13. Calverly PMA, Barnes PJ. Pro/Con Editorials. Am J Respir Crit Care Med 2000; 161:341-4.
14. Chanez PA, Vignola M, O'Shaugnessy T et al. Corticosteroid reversibility in COPD is related to features of asthma. Am J Respir Crit Care Med 1997; 155:1529-34.

B. PROCEDURES

Detailed procedures [ix]

Outline of Protocol:

•This will be a double-blind, placebo controlled, parallel group study.

• Subjects will be randomised 2:1 to receive active medication (Fluticasone 500 mcg bd) or placebo bd, both given in the same way via identical dry powder, "Diskus" devices for a duration of 6 months.

• After recruitment, but before initiation of randomised therapy, each patient will undergo:

Full spirometry with a flow-volume loop, before and after 200 mcg inhaled salbutamol (a β agonist bronchodilator) given via a spacer device.

A Quality Of Life Questionnaire (QOL) (St George's Respiratory Questionnaire) will be completed.

Induction of sputum (by spontaneous coughing or by our standard laboratoryo method of nebulisation of hypertonic saline, if necessary).

At least 48 hours later, standard flexible fibreoptic bronchoscopy will be performed under local anaesthetic, with 6 endobronchial biopsies being taken from around the orifices of middle or lower lobe bronchi, and a standard 3 X 60 ml bronchoalveolar lavage (BAL) will be performed. Before bronchoscopy, 200 mcg of salbutamol and 80 mcg of ipratropium bromide will be given by inhaler. Supplemental oxygen will be given throughout the procedure and oxygen saturation will be monitored using a finger probe from a pulse oximeter.

• At the end of the study period, these four procedures will be repeated ie questionnaire, pulmonary function, sputum collection and bronchoscopy (with the latter at least 48 hours later to avoid any confounding by potential acute cellular changes induced by sputum induction). Study medication will continue until these investigations are complete.

Study Visits

Recruitment (Visit 1)

This will be via our volunteer database for smokers or ex-smokers with some respiratory symptoms.

- A detailed respiratory and medical history will be taken and physical examination performed.
- An ECG will be performed.
- Spirometry will be undertaken.
- Inclusion and Exclusion criteria will be assessed. If appropriate, the study will be explained in detail, and the explanatory leaflet will be read and discussed before written consent is obtained.
- A blood sample will be obtained for routine full blood count, U&E and creatinine, liver function tests and random glucose.

First Study Visit (Visit 2) usually within 2 weeks of recruitment and following at least 1 week of run-in during which a symptom diary card will be maintained to collect data on daily symptom scores and sputum produced.

- The subject's clinical stability will be checked.
- The QOL (St GRQ) will be delivered, spirometry performed.
- Sputum will be collected and sent to the research lab for processing.

- A bronchoscopy (Visit 3) will then be arranged within approximately one week, but at least 48 hours later.

- Study medication will then be given to the patient according to a randomisation schedule held in pharmacy.

Intermediate check visits (Visits 4 and 5), at 2 month and at 4 months. The subject will be asked to attend the laboratory every 8 weeks (± 2 weeks) to assess well being and check on compliance with study medication. Occurrence of any exacerbations will be recorded. New inhaler devices will be prescribed.

If an exacerbation were to occur in the intervening time, the patient will be asked to contact the Research Laboratory. These exacerbations occur commonly in COPD and are defined as worsening of symptoms requiring additional medication, either with an antibiotic or oral

Prednisolone (a corticosteroid) or both. The patient will be given advice about the best course of action – either to visit the General Practitioner, or if the patient prefers to attend the research laboratory for medical review.

Final visit

Two weeks before the expected completion of the study, each subject will be contacted by phone to check on wellbeing, and will then be sent a diary card to collect data over 1 week on daily symptoms and sputum production. At the end of 6 months (Visit 6) of study medication, each subject will return to the laboratory for repeat QOL questionnaire, spirometry and sputum collection, and after a period of at least 48 hours, but within approximately 1 week bronchoscopy will be performed, (Visit 7) as before. Any exacerbations in the previous 2 months will be recorded.

Study medication will be ceased. Approximately 1 week later the patient will be phoned to check on well being, and if there is any deterioration a visit to the laboratory will be arranged at the patient's convenience, for full clinical review.

Study outcome measures/efficacy measurements

- Changes in the QOL measure and spirometric values (large and small airway flow rates)
- Sputum neutrophil and eosinophil percentage counts
- Bronchoalveolar lavage cell differential counts and FACS anlaysis (lymphocyte and macrophage subtypes and activation markers). BAL supernatant will be stored for cytokine and chemokine assays and assay of selected mediators, eg ECP, neutrophil elastase and myeloperoxidase.
- Airway biopsies: the initial emphasis will be on acute and chronic cellular inflammation: numbers of eosinophils, neutrophils, lymphocytes and macrophages and activation markers HLADR and CD25 but with the potential for later study of indices of scarring and "remodelling" of the airway wall ie collagen I, III and V deposition, epithelial integrity, subepithelial basement membrane thickness and degree of vascularity in the subepithelial lamina propria area (by collagen IV staining).

NB Over the past 15 years of research into airway pathology using fibre-optic bronchoscopy, we have developed a wide range of end points with which to assess both BAL and biopsy material. The degree to which we can fully utilise the material will depend upon funding over the next three years. However, we already have sufficient funding to allow assessment of acute and chronic cellular infiltrates in the airway, which is our primary pathological endpoint. Further grant applications will be developed over the next 12-18 months, based upon this study protocol, the extremely valuable biological material obtained and our extensive "track record" in this field of research (see references).

Statistical Analysis

At the end of the study we will intend to have 24 paired sets of data in volunteers treated with active intervention (Fluticasone) and 12 in placebo treated controls.

There will be 4 main approaches to analysis:

- Baseline bronchoscopic data as a whole will be analysed against our current historic normal control data, of which we have large numbers both for airway biopsy (n=24) and BAL (n=36). This will give us excellent power to detect even subtle changes within COPD cell profiles (and later structural components).
- The placebo control material will be used to establish stability over time in biological signals and to establish coefficients of repeatability for cellular indices. Paired analysis within the actively treated group will be undertaken to indicate which cellular populations or activation markers may be susceptible to change with ICS, and then changes over time in these indices will be compared with respective changes in the placebo treated control material. We have previously published repeatability data in chronic stable asthmatics, and done similar calculations in post lung transplant airway samples, which indicate that optimal power for detecting changes with time is achieved with approx. 15 data pairs for most cell lines. With the numbers available in this COPD study, we therefore have substantial "reserve".
- The central analysis will focus on the ICS treated group and use changes over time in both cellular indices (in BAL and biopsies) and clinical and physiological end points, and undertake regression analyses to define which, if any, of the cellular changes are related to clinical and/or physiological improvements.

- **We will perform post-hoc analyses in selected sub-groups, namely:**
  - **a) those in whom regular sputum production is a feature.**
  - **b) those with above normal airway eosinophils.**
  - **c) those with more or less severe lung function abnormalities (best and worse 10) to define whether ICS have more benefit in these patients than in COPD in general.**

This will be a large and complex data set, but we have extensive experience in relating clinical, physiological and bronchoscopic pathological end points. We have developed close collaboration with Mr Michael Bailey and Dr Andrew Forbes, our statistical advisers at Monash University, and they will again by providing assistance with this study.

SUBJECTS

Selection of subjects [x]

Inclusion criteria:

- Age 40-70 years
- 36 subjects with smoking-related COPD to complete the study, which will mean likely need for initial recruitment of up to 45 patients (both male and female will be included).
- Minimum of 15 pack-year history of cigarette smoking. Either current or ex smokers.
- FEV1 40% to 80% predicted and/or FEF50<50% with FER (ratio of FEV1 to FVC) ≤70% post bronchodilator and definite scalloping out of the descending limb of the flow-volume loop on spirometry. Absolute FEV ≥ 1.5 litres.
- Bronchodilator therapy with a short acting beta agonist prn and anticholinergics in stable doses will be allowable.

Exclusion criteria:

- A history of asthma, either by doctor's diagnosis or with classic clinical features ie episodic wheezing relieved by bronchodilator, typical diurnal variation of symptoms of chest tightening, wheeze and breathlessness, and these classic symptoms precipitated by specific exposure to "trigger factors" such as dust, fumes and exercise. If there is doubt about diagnosis, this will be decided through consensus by at least 2 of the clinician investigators.
- A history of symptomatic cardiovascular disease or significant renal/hepatic disease or diabetes mellitus. An ECG will be performed and must lack ischaemic changes.
- Warfarin therapy and/or a low platelet count (< 120).
- Use of oral corticosteroid or ICS medication in last 3 months before recruitment.
- Use of long acting β agonist in past 4 weeks.
- Upper respiratory tract infection or acute exacerbation in past 4 weeks.
- Oxygen saturation ≤ 92% at rest i.e.: excluding ventilatory failure.

Recruitment of subjects [xi]

For the RHH limb of this study, subjects who have previously been involved in smoking cessation studies, or specifically in studies of COPD and who are on the Dept of Respiratory Medicine database of volunteers will be approached by phone or letter. In Melbourne, recruitment was by advertsiement in the press or in public places, but it is not envisaged at this stage that such strategies will be necessary in Tasmania.

Information about subjects

(i) State whether information will be identified, potentially identifiable or unidentified. [xii]
All individuals will be identified to the sponsor by initials and identification number only. Patients will be informed in the plain language statement that their hospital based research record may be viewed by the sponsor or other regulatory bodies – but for source verification only.

(ii) State source(s) of information. [xiii]
Information will be available from records of previous clinical and physiological assessments held by the Dept of Respiratory Medicine.
(iii) Will data on individual subjects be obtained from any Commonwealth Government agency? [xiv]
If so, name agency. NO.

Potential risks [xv]<br>**The main issue in this study is undertaking bronchoscopy in a volunteer population. However, Prof Walters's research team has a long track record of bronchoscopic reseearch in asthma, lung transplant recipients, and more recently in COPD patients. Overall, Prof Walters has been CI and had overall responsibility for approx. 600 research bronchoscopic procedures in the past 15 years.**<br><br>**In terms of lung function, this group of COPD patients will be comparable to or even less impaired than many subjects studied over the past 15 years. We will not be including any patient with an FEV_1 of < 1.5 litres, which is likely to be > 50% predicted in all subjects. Subjects will be premedicated with β-agonist and anticholinergic bronchodilators and oxygen saturation will be monitored. Supplemental oxygen will be given routinely.**
Post contact [xvi]<br><br>**Patients are to be phoned 1 week after final cessation of study medication to check on clinical stability. If there has been any suggestion of deterioration, the individual will be invited to the RHH for clinical review.**
Remuneration [xvii]<br><br>Subjects will be reimbursed travel expenses, and it has been our policy in such studies to offer $100 for compensation for time and discomfort for each bronchoscopy.
Confidentiality and anonymity [xviii]<br><br>All documents pertaining to the study will be securely locked in the Dept of Respiratory Medicine research room during the period of the study, and available only to the research team. They will then be kept for 15 years, after which time they will be destroyed under supervision. Due to space constraints it is likely that the documents will be archived outside the hospital by the sponsor, but with access limited to the CI only. There will be no electronic data collected for this study.
Administration of substances/agents [xix]<br><br>Fluticasone propionate, 500 mcg bd, or identical placebo medication, will be taken by the patients from a dry powder "Diskus" device for a duration of treatment of 6 months. This is a fully licenced inhaled corticosteroid with an excellent safety profile at this dose and taken in this way.
Human tissue or body fluid sampling [xx]<br><br>1. Sputum, either produced spontaneously or induced by hypertonic saline inhalation in a standard way. This is processed to provide smears on slides for cell counting and separation of the liquid component for storage and future analysis of mediators.<br>2. Bronchoalveolar lavage (3X60 ml aliquots) for analysis of cells and solutes.<br>3. 6 endobrachial biopsies taken with cupped forceps (1-2 mm^3) for tissue processing for analysis of inflammatory cells, fibrosis and angiogenesis using image analysis.
Other ethical issues [xxi]<br><br>At the end of the clinical study, hopefully at the end of 2002, we will transfer all the archived biopsies and slides (BAL and sputum) and stored sputum and BAL supernatants to Hobart from Melbourne for further analysis. We will eventually wish to perform comprehensive analysis of these samples for inflammation and markers of airway scarring and remodelling. This work will form the basis of an NHMRC grant application in 2003

Information sheet [xxii]

Attached.

Consent form [xxiii]

Attached.

C. DECLARATIONS

Statement of scientific merit [xxiv]

The *Head of School** is required to sign the following statement:
This proposal has been considered and is sound with regard to its merit and methodology.

A. CARMICHAEL	[signature]	6/12/01
(Name of Head of School)	(Signature)	(Date)

* In some schools the signature of the Head of Discipline may be more appropriate.
* The certification of scientific merit may not be given by an investigator on the project.

Conformity with NHMRC guidelines [xxv]

The *chief investigator* is required to sign the following statement:
I have read and understood the *National statement on ethical conduct in research involving humans 1999.* I accept that I, as chief investigator, am responsible for ensuring that the investigation proposed in this form is conducted fully within the conditions laid down in the *National Statement* and any other conditions specified by the University Human Research Ethics Committee.

E H WALTERS	[signature]	4.12.01
(Name of chief investigator)	(Signature)	(Date)

Conformity with code of practice: human tissue and body fluid sampling

The *chief investigator* is required to sign the following statement in relation to relevant research projects/teaching exercises:
I have read the Human Research Ethics Committee *Code of Practice: Human Tissue and Body Fluid Sampling* and confirm that this Code will be followed.

E H WALTERS	[signature]	4.12.01
(Name of chief investigator)	(Signature)	(Date)

Signatures of other investigators [xxvi]

R WOOD-BAKER	[signature]	5/12/01
(Name)	(Signature)	(Date)

Relevant references
List references
(a) by the investigator; The References from Prof Walters work are to emphasise experience in such bronchoscopically based studies in airway disease.

Book Chapters.

1. E.H Walters, H. Booth & D.P. Johns. Clinical Investigation of Interstitial Lung Disease. *In* Immunology and Management of Interstitial Lung Disease. E.H. Walters and R. DuBois (Eds). Chapman and Hall **1995**; 37-60.
2. T.Kotsimbos & E.H. Walters. Bronchoscopy, Lavage, Needle and Other Biopsies. *In* Medicine International. M Muers and J. Hopkin (Eds). **1995**; 23: 250-256.
3. Ward C, Walters E H. Bronchoalveolar Lavage (BAL); Critical Evaluation of Techniques. In: Measurement of Human Airway Inflammation (Methods in Molecular Medicine Series). Rogers DF, Donnelly LE (Eds). *Chapman and Hall.* **2001**; (In Press)

Papers

1. H. Booth, I. Richmond, C. Ward, R. Harkawat, E.H. Walters. Effect of High Dose Fluticasone Propionate in Airway Inflammation in Asthma. *Am. J. Respir. Crit. Care Med.* **1995**; 152:45-52.
2. Ward C, Gardiner P.V., Booth H, Walters E.H. Intrasubject Variability in Airway Inflammation Sampled by Bronchoalveolar Lavage in Stable Asthmatics. *Eur. Respir. J.* **1995**; 8: 1866-71.
3. Richmond, H. Booth, C. Ward, E.H. Walters. Intrasubject Variablity in Airway Inflammation in Biopsies in Mild to Moderate Stable Asthma. *Am. J. Resp. Crit. Care Med.* **1996**; 153:899-903.
4. Tang C, Rolland J, Ward C, Bish R, Thien F, Walters EH. Seasonal Comparison of Cytokine Profiles in Atopic Asthmatics and Atopic Non-Asthmatics. *Am. J. Resp. Crit. Care Med.* **1996**; 154: 1615-22.
5. Walters EH, Ward C, Li X. Bronchoalveolar Lavage in Asthma Research (Review article). *Respirology.* **1996**; 1:233-45.
6. Tang C, Rolland JM, Ward C, Quan B, Walters EH. Allergen-Induced Airway Reactions in Atopic Asthmatics Correlate with Allergen-Specific IL-5 Response by BAL Cells. *Respirology.* **1997**; 2: 45-55.
7. Tang C, Rolland JM, Ward C, Quan B, Walters EH. IL-5 Production by Broncho-alveolar Lavage and Peripheral Blood Mononuclear Cells in Asthma and Atopy. *Eur. Respir. J.* **1997**; 10: 624-632. (Plus accompanying Editorial)
8. Snell GI, Ward C, Wilson JW, Orsida B, Williams TJ, Walters EH. Immunopathological Changes in the Airways of Stable Lung Transplant Recipients. *Thorax.* **1997**; 52: 322-328.
9. Zheng L, Ward C, Snell GI, Orsida BE, Li X, Wilson JW, Williams TJ, Walters EH. Scar Collagen Deposition in the Airways of Allografts of Lung Transplant Recipients. *Am J Respir Crit Care Med.* **1997**; 52: 551-556.
10. Liakakos P, Snell GI, Ward C, Johns DP, Bamford TL, Williams TJ, Walters EH. Bronchial Hyper- Responsiveness in Lung Transplant Recipients - Lack of Correlation to Airway Inflammation. *Thorax.* **1997**; 52: 551-556.
11. Ward C, Fenwick J, Booth H, Walters EH. Albumin is not suitable as a marker of Bronchoalveolar Lavage (BAL) dilution in Interstitial Lung Disease (ILD). *Eur Respir J.* **1997**; 10: 2027-2033.

12. Kotsimbos ATC, Sinickas V, Glare EM, Esmore DS, Snell GI, Walters EH, Williams TJ. Quantitative Detection of Human Cytomegalovirus DNA in Lung Transplant Recipients. *Am J Respir Crit Care Med.* **1997**; 156: 1241-1246.
13. Ward C, Snell G.I, Zheng L, Orsida B, Whitford H, Williams T.J, Walters E.H. Endobronchial biopsy and BAL in clinically stable lung transplant recipients and chronic rejection. *Am. J. Respir. Crit. Care Med.* **1998;** 158: 84-91.23. Ward C, Snell G.I, Orsida B, Zheng L, Williams T.J, Walters E.H. Airway versus transbronchial biopsy and BAL in lung transplant recipients: different but complementary. *Eur. Respir. J.* **1997**; 10: 2876 - 2880.
14. Ward C, Snell G.I, Zheng L, Orsida B, Whitford H, Williams T.J, Walters E.H. Endobronchial biopsy and BAL in clinically stable lung transplant recipients and chronic rejection. *Am. J. Respir. Crit. Care Med.* **1998;** 158: 84-91.
15. Tang C, Rolland JM, Li X, Ward C, Bish R, Walters EH. Alveolar macrophages from atopic asthmatics, but not atopic non asthmatics enhance IL-5 production by CD4+ Tcells. *Am J Respir Crit Care Med* **1998** ;157: 1120-1126
16. Tang C, Rolland JM, Ward C, Thien F, Li X, Gollant S, Walters EH. Differential regulation of allergen-specific Th2- but not Th1-type responses by alveolar macrophages in atopic asthma. *J. Allergy Clin Immunol* **1998** ; 102: 368-375
17. Zheng L, Orsida BE, Ward C, Wilson JW, Williams TJ, Walters EH, Snell GI. Airway vascular changes in lung allograft recipients. *J Heart Lung Transplantation.* **1999;** 18: 231-238.
18.. Orsida BE, Li X, Hickey B, Thien FC, Wilson JW, Walters EH. Submucosal vascularity in asthmatic airways: Relation to inhaled steroid dose. *Thorax* **1999;** 54: 289-295. (Plus accompanying editorial by Jim Hogg).
19. Tang C, Rolland JM, Ward C, Li X, Bish R, Thien F,and Walters EH. Modulating effects of alveolar macrophages on CD4 + T cell IL-5 responses correlate with IL-1β, IL-6, and IL-12 production. *Eur. Respir. J* **1999;** 14: 106-12.
20. Li X, Ward C, Bish R, Bamford T, Thien F, Wilson JW, Walters EH. An anti-Gabbay E, Walters EH, Orsida B, Whitford H, Ward C, Kotsimbos TC, Snell GI, Williams TJ. In stable lung transplant recipients, exhaled nitric oxide levels positively correlate with airway neutrophilia and bronchial epithelial iNOS. *Am J Resp Crit Care Med.* **1999;** 160:2093-2099
21. Glare EM, Divjak M, Rolland JM, Walters EH. Asthmatic airway biopsy specimens are more likely to express the IL-4 alternative splice variant IL-4δ2. *Journal of Allergy and Clinical Immunology* 1999; 104: 978-982.
22. Ward C, Effros RM, Walters EH. Assessment of epithelial lining fluid dilution during bronchoalveolar lavage. *European Respiratory Review* 1999; 6, 66:32-37.
23. Zheng L, Walters EH, Wang N, Orsida B, Ward C, Whitford H, Williams TJ, Gabbay E, Walters EH, Orsida B, Whitford H, Ward C, Kotsimbos TC, Snell GI, Williams TJ. Post lung transplant Bronchiolitis Obliterans Syndrome (BOS) in characterised by increased exhaled nitric oxide levels and epithelial inducible nitric oxide synthase. *Am J Resp Crit Care Med.* **2000;** 162: 2194-2200.
24. Whitford H, Orsida B, Kotsimbos T, Pais M, Ward C, Zheng L, Williams T, Walters EH, Snell G. Bronchoalveolar lavage cellular profiles in lung transplantation: the effect of inhaled corticosteroid. *Annals of Transplantation* **2000**; 5: 32-7.
25. Walters EH, Bjermer L, Faurschou P, sandstrom T. The anti-inflammatory profile of inhaled corticosteroids combines with Salmeterol in asthmatic patients. *Respiratory Medicine* **2000**; 94: SuppF 26-31.
26. Tang C, Ward C, Reid D, Bish R, Walters EH. Normally suppressing CD40 86. Orsida BE, Ward C, Li X, Bish R, Wilson JW, Thien F, Walters EH. Effect of a

long acting beta agonist over three months on airway wall vascular remodelling in asthma. *Am J Respir Crit Care Med.* **2001;** 164: 117-21.

27. Glare C, Divjiak M, Bailey M, Walters EH. The usefulness of competitive PCR: airway gene expression of IL–5, IL-4, IL-4delta2,IL-2 and IFNgamma in asthma. *Thorax.* **2001;** 56:541-8.
28. Orsida BE, Ward C, Li X, Bish R, Wilson JW, Thien F, Walters EH. Effect of a long acting beta agonist over three months on airway wall vascular remodelling in asthma. *Am J Respir Crit Care Med.* **2001;** 164: 117-21.
29. Reid D, Snell GI, Ward C, Krishnaswamy r, Ward R, Zheng L, Williams T, Walters EH. Iron overload and nitric oxide-derived oxidative stress following lung transplantation. *J Heart Lung Transplantation.* . **2001.** (Accepted).
30. Ward C, Whitford H, Snell, GI Bao H, Zheng L, Kotsimbos ATC, Reid Dw, Williams TJ, Walters EH. Bronchoalveolar Lavage Macrophage and Lymphocyte Phenotypes in Lung Transplant Recipients. *Internatioaln J Heart Lung Transplantation.* . **2001.** (Accepted).
31. Reid D, Walters EH, Johns DP, Whitford H, Ward C, Khov S, Liakakos P, Reid S, Williams TJ, Snell G. Hypertonic Saline Challenge Predicts Early Onset Bronchiolitis Obliterans Syndrome Post-Lung Transplantation. *Annals of Transplantation.* . **2001.** (Accepted).
32. Whitford HM, Orsida B, Paid M, Levvey B, Ward C, Reid S, Reid D, Williams TJ, Kotsimbos T, Walters EH, Snell GI. Bronchoalveolar Lavage (BAL) Neutrophilia in Lung Transplant Recipients (LTR), Infection and Bronchiolitis Obliterans Syndrome (BOS), the Chicken or the Egg? *Annals of Transplantation.* . **2001.** (Accepted).
33. Whitford HM, Orsida B, Paid M, Levvey B, Ward C, Williams TJ, Kotsimbos T, Walters EH, Snell GI. Features of Bronchoalveolar Lavage (BAL) in Lung Transplant Recipients (LTR) who Later Develop Bronchiolitis Obliterans Syndrome (BOS). *Annals of Transplantation.* **2001.** (Accepted).
34. Ward C, Johns DP, Bish R, Pais M, Reid DW, Ingram C, Walters EH. Reduced airway distensibility, fixed airflow limitation and airway wall remodelling in asthma. *Am J Respir Crit Care Med.* **2001.** (Accepted).
35. Ward C, Pais M, Bish R, Reid D, Feltis B, Walters EH. Airway inflammation, basement membrane thickening and bronchial hyper-responsiveness in asthma. *Thorax.* **2001**; (Accepted).
36. Orside BE, Krozowski ZS, Walters EH. Clinical relevance of airway II β-hydroxysteroid dehydrogenase type II enzyme in asthma-relation to inhaled corticosteroid needs. *Am J Respir Crit Care Med.* . **2001**; (Accepted).
